# Porcine small intestinal organoids as a model to explore ETEC–host interactions in the gut

**DOI:** 10.1186/s13567-021-00961-7

**Published:** 2021-06-26

**Authors:** Bjarne Vermeire, Liara M. Gonzalez, Robert J. J. Jansens, Eric Cox, Bert Devriendt

**Affiliations:** 1 Department of Virology, Parasitology, Immunology, Faculty of Veterinary Medicine, Laboratory of Immunology, Ghent University, 9820 Merelbeke, Belgium; 2 Laboratory of Intestinal Regenerative Medicine, College of Veterinary Medicine, NCSU, Raleigh, NC USA

**Keywords:** Intestinal stem cells, Enteroids, ETEC, Swelling assay, Pig

## Abstract

Small intestinal organoids, or enteroids, represent a valuable model to study host–pathogen interactions at the intestinal epithelial surface. Much research has been done on murine and human enteroids, however only a handful studies evaluated the development of enteroids in other species. Porcine enteroid cultures have been described, but little is known about their functional responses to specific pathogens or their associated virulence factors. Here, we report that porcine enteroids respond in a similar manner as in vivo gut tissues to enterotoxins derived from enterotoxigenic *Escherichia coli*, an enteric pathogen causing postweaning diarrhoea in piglets. Upon enterotoxin stimulation, these enteroids not only display a dysregulated electrolyte and water balance as shown by their swelling, but also secrete inflammation markers. Porcine enteroids grown as a 2D-monolayer supported the adhesion of an F4^+^ ETEC strain. Hence, these enteroids closely mimic in vivo intestinal epithelial responses to gut pathogens and are a promising model to study host–pathogen interactions in the pig gut. Insights obtained with this model might accelerate the design of veterinary therapeutics aimed at improving gut health.

**Supplementary Information:**

The online version contains supplementary material available at 10.1186/s13567-021-00961-7.

## Introduction

Enterotoxigenic *E. coli* (ETEC) are a major cause of postweaning diarrhoea in piglets and result in severe economic losses in swine husbandry due to increased mortality, reduced growth rates and elevated medication [1]. Estimates range from EUR 40–314 per sow depending on the severity of the disease [2, 3]. Upon ingestion, ETEC reach the small intestine and adhere to the epithelium through the interaction of its fimbriae with fimbrial receptors on the apical membrane of the enterocytes. The most prevalent fimbriae expressed by ETEC causing postweaning diarrhoea are F4 and F18 fimbriae, which mainly bind to diverse glycoproteins, such as aminopeptidase N and fucosyl-containing glycosphingolipids, respectively [4, 5]. This results in the secretion of heat-labile (LT) and heat-stable (STa, STb) enterotoxins, which disrupt the water and electrolyte balance, ultimately leading to the watery diarrhoea characteristic of ETEC infections [6, 7]. In piglets, the enterotoxin STb plays an important role in eliciting diarrhoea during the acute phase of the infection. In turn, the host intestinal tissues, including the small intestinal epithelium, respond to ETEC infection by producing pro-inflammatory mediators, such as interleukin-8 (IL8, CXCL8) and IL6, to enable a rapid induction of innate immune responses and subsequent protective secretory IgA responses [8–10].

Despite this progress in understanding the molecular pathogenesis of ETEC in piglets, many facets, such as the impact of ETEC and its enterotoxins on the function of intestinal epithelial cells, remain unresolved. This is primarily due to a lack of effective in vitro and in vivo models. Available cell lines fail to represent the cellular complexity and functionality of the epithelium. Explants such as precision cut intestinal sections are an alternative model, however these are only applicable for short term experiments [11]. Animal experiments such as ligated loops or perfused intestinal segments on the other hand are quite labour-intensive and pose a range of technical difficulties [12]. Hence, there is a need for more complex in vitro based research, such as the recently developed techniques for the culture of primary intestinal epithelial stem cells. This system allows for the development of complex three-dimensional intestinal epithelial structures grown in a laminin/collagen-rich hydrogel (Matrigel), called enteroids. Utilization of enteroids for research has dramatically increased since the discovery of Lgr5^+^ intestinal stem cells and the development of human and murine culture techniques [13]. A major advantage of these enteroid cultures over classic intestinal epithelial cell lines is the confirmed presence of the different epithelial cell lineages, making it a much more representative model for the in vivo situation [14]. Furthermore, enteroids, which are grown from isolated intestinal crypts, retain segment specificity, a feature of particular interest for host–pathogen interaction studies [15, 16]. However, in contrast to human and murine studies, literature on porcine enteroids is scarce. The development and cellular composition of the porcine enteroids has been described [14, 17–20], but to the best of our knowledge functional responses of enteroids towards porcine pathogens have only recently been reported [21–25]. Here, we build further on previous studies to report the development of porcine enteroids from all segments of the small intestine in different culture media, the generation of enteroid monolayers and focus on the functional responses elicited by ETEC-derived enterotoxins.

## Materials and methods

### Bacterial cultures and enterotoxin production

To produce the ETEC enterotoxins (mixture of LT, STa and STb), the GIS26 ETEC reference strain and an isogenic enterotoxin knock out strain (GIS26*ΔestAΔestB:KAN*; toxin negative strain) were grown as described [8]. Briefly, single colonies on a brain heart infusion (BHI) agar plate were grown in CAYE medium at 37 °C while shaking at 200 revolutions per minute (rpm) for 24 h. Next, the bacteria were cleared from the culture medium with two centrifugation steps (5000 *g*; 20 min; 4 °C). After the second centrifugation step, the supernatant was filtered with a 0.22 µm low protein binding filter (Novolab, Geraardsbergen, Belgium). This sterile solution was aliquoted and stored at −20 °C for later use.

### Wnt, R-spondin and Noggin (WRN) conditioned medium

The L-WRN cell line (ATCC, Manassas, VA, USA), derived by transfecting the mouse L-Wnt3a fibroblast-like cell line with an R-spondin 3 and noggin co-expressing vector was maintained following the ATCC guidelines in Dulbecco’s modified Eagle’s medium (DMEM, Gibco, Waltham, MA, USA) supplemented with 10% FBS (Sigma, St. Louis, MO, USA); 0.5 mg/mL Geneticin (G-418; Gibco); 0.5 mg/mL hygromycin B (Invitrogen, Carlsbad, CA, USA) at 37 °C, 5% CO_2_ and 90% humidity. At 90% confluency the cells were washed with sterile phosphate buffered saline [PBS, room temperature (RT)], detached with trypsin buffer (0.25% trypsin (Gibco), 100 U/mL penicillin, 100 μg/mL streptomycin and 1% Versene in PBS) for 10 min at RT and passaged to new culture flasks at a 1:10 ratio.

To prepare L-WRN conditioned medium, L-WRN cells were cultured with cell culture medium lacking G-418 and hygromycin B (collection medium) as described previously [26]. The cell culture medium was replaced every other day until the cells became fully confluent (usually 3–4 days). The collection medium was then refreshed and collected after incubating the cells for another 24 h. This was repeated three times. The collected medium was centrifuged at 3000 *g*, 4 °C for 15 min to remove any remaining cells, mixed with an equal volume of intestinal epithelial stem cell (IESC; see below) medium to produce L-WRN conditioned (50%) IESC medium, aliquoted and stored at −20 °C.

### Isolation of small intestinal crypts

Small intestinal crypts were isolated from 6- to 10-week-old piglets as previously described with minor modifications [14, 27]. After euthanasia, the abdominal cavity was opened and the small intestine was located. Ten cm sections of duodenum, jejunum without Peyer’s patches and ileum were isolated and immediately put in cold, sterile PBS. Each piece was rinsed well in PBS supplemented with 100 U/mL penicillin and 100 μg/mL streptomycin to wash away intestinal contents. Sections of ~ 5 cm were inverted and then tied to a wooden swab of similar length using surgical suture wire. The ileal tissue was processed to remove the Peyer’s patches. The intestinal tissues were then incubated in 35 mL cold dissociation buffer 1 (30 mM Ethylenediaminetetraacetic acid (EDTA, VWR, Radnor, PA, USA), 1.5 mM dithiothreitol (Sigma), 6 µM Rho-associated kinase (ROCK) inhibitor (Y-27632; Sigma) in PBS) for 30 min on ice on an orbital shaker. Every 5 min the tissues were shaken vigorously for 10–15 s. After 30 min the samples were transferred to 35 mL warm (37 °C) dissociation buffer 2 (30 mM EDTA, 6 µM Y-27632 in PBS) for 10 min on an orbital shaker. Next, the tissue was transferred to cold, sterile PBS and incubated for 5 min, while the shaking frequency was increased to every 1–2 min. To assess crypt detachment and purity, a droplet of this suspension was examined under a light microscope. After 5 min the tissue samples were transferred to fresh, cold PBS and the above steps were repeated until single crypts with minimal debris were present. Upon choosing the best fraction, crypts were counted, centrifuged at 200 *g* for 5 min and then resuspended in 1 mL cold, sterile PBS.

### Three-dimensional enteroid culture

The isolated crypts were centrifuged at 200 *g*, 4 °C for 5 min and were resuspended on ice in Matrigel (growth factor reduced, phenol red free, Corning, Corning, NY, USA) containing a growth factor mix (5.5 µg/mL recombinant (rec) human (hu) R-spondin (R&D Systems, Minneapolis, MN, USA), 1.65 µg/mL rec hu Noggin (Peprotech, Rocky Hill, NJ, USA), 1.65 µg/mL rec hu Wnt3a (R&D Systems), 825 ng/mL rec hu epidermal growth factor (EGF) (R&D Systems), 8.25 µM A83-01 (Tocris Bioscience, Bristol, UK), 50 µM SB202190 (Sigma), 16.5 mM nicotinamide (Sigma), 165 nM Gastrin (Sigma), 165 µM Y-27632 (Sigma), 8.25 µM LY2157299 (Selleckchem, Houston, TX, USA), 41 µM CHIR99021 [Cayman Chemicals, Ann Arbor, MI, USA)]. Per well a 50 µL droplet of the Matrigel containing 75 crypts was slowly brought onto a pre-warmed (37 °C) 24-well plate, allowing the formation of a small dome in the centre of the well. Plates were placed at 37 °C for 30 min to allow the Matrigel to polymerize upon which 500 µL of IESC medium (Advanced Dulbecco’s modified Eagle’s medium/Nutrient mixture F-12 (Gibco), 1 × N-2 supplement (Gibco), 1 × B-27 Supplement (Gibco; no Vit A), 10 mM 4-(2-hydroxyethyl)-1-piperazineethanesulfonic acid (HEPES, Gibco), 1% Glutamax (Gibco) and 1% P/S) supplemented with 500 ng/mL R-spondin, 100 ng/mL Noggin, 100 ng/mL Wnt3a, 50 ng/mL EGF, 0.5 µM A83-01, 10 µM SB202190, 10 mM nicotinamide, 10 nM Gastrin, 10 µM Y-27632, 0.5 µM LY2157299 and 2.5 µM CHIR99021. Crypts were subsequently cultured at 37 °C, 5% CO_2_ and 90% humidity. After 2 days fresh growth factor mix was added to the cultures in a similar concentration as the complete IESC medium and another 2 days later the complete IESC medium was replaced. For most experiments, crypts were cultured in L-WRN-conditioned IESC medium supplemented with 50 ng/mL EGF, 0.5 µM A83-01, 10 µM SB202190, 10 mM nicotinamide, 10 nM Gastrin, 10 µM Y-27632, 0.5 µM LY2157299 and 2.5 µM CHIR99021. Alternatively, enteroids were grown using Intesticult organoid growth medium (human, Stemcell Technologies). Every 2 days this culture medium was replaced. This procedure was repeated until enteroids were passaged.

Matrigel domes were washed twice with cold, sterile PBS, followed by addition of 0.5 mL cold cell recovery solution (Corning). This was done forcefully to break up the dome. Plates were incubated for 30 min on ice and the enteroids were collected, centrifuged (200 *g*, 5 min, 4 °C) and resuspended in 1 mL cold PBS containing 10 µM Y-27632. The enteroids were then fragmented by passing them twice through a 27 Gauge needle with a syringe. A fraction of the fragments was spun down (200 *g*, 5 min, 4 °C), resuspended in 50 µL Matrigel containing the growth factor mix mentioned above, plated on a pre-warmed (37 °C) 24-well plate and placed at 37 °C for 30 min to allow polymerisation. Next, 500 µL complete IESC, L-WRN-conditioned IESC or Intesticult medium was added to each well. Enteroid development from small intestinal crypts and upon passage was evaluated using an Olympus IX81 light microscope (Olympus, Tokyo, Japan).

### Monolayers

Microtiter wells were coated with 2.5 μg/cm^2^ collagen IV (mouse, corning) for 1 h at RT, washed twice with PBS and air-dried. Fragmented enteroids obtained during passage were resuspended in medium and plated on collagen IV coated wells. Alternatively, further dissociation was achieved by incubation with 0.25% trypsin in PBS at 37 °C for 10 min while repeatedly pipetting. Cells were then plated at a concentration of 25 000–40 000 cells/cm^2^. Confluence was achieved after 4–7 days depending on the growth medium.

### SOX9 staining

The presence of SOX9, a marker for progenitor and stem cells [28], in enteroid cultures was evaluated by immunocytochemistry. Enteroids were washed twice with sterile PBS and subsequently fixated for 30 min with 4% paraformaldehyde at RT. After each incubation step, cells were repeatedly washed twice with sterile PBS. Upon fixation the enteroids were treated with ammonium chloride (50 mM in PBS) for 30 min at RT and then permeabilized with 0.1% Triton X-100 in PBS again for 30 min at RT. Next, the enteroids were blocked with a 5% BSA solution for 1 h at RT and the rabbit anti-SOX9 antibody (Millipore, Burlington, MA, USA) or irrelevant rabbit IgG (both at 1 µg/mL in PBS + 0.1% goat serum) was added overnight at 4 °C. Before adding the Fluorescein isothiocyanate conjugated anti-rabbit IgG (Sigma; 1/100 dilution in PBS), the wells were washed five times with sterile PBS. Next, the cells were incubated for 2 h at RT with the secondary antibody and subsequently washed 5 times. The nuclei were counterstained with Hoechst (10 µg/mL) for 10 min at RT, the enteroids were covered with mounting liquid and were imaged with an Olympus IX81 fluorescence microscope. Images were processed with ImageJ.

### Swelling assay

Enteroid fragments were cultured in Matrigel until spheroids or less complex enteroids developed. At that developmental stage, the enteroid cultures were washed with IESC medium (500 µL/well; 37 °C) and subsequently, cell culture medium was added containing guanylin (10 µM) or bacterial culture supernatant (1/20 dilution) with or without a mixture of the ETEC enterotoxins (LT, STa and STb), produced by the GIS26 ETEC reference strain or the isogenic enterotoxin knock out strain (GIS26*ΔestAΔestB:KAN*; toxin negative strain), respectively. The enteroids were then monitored during two hours using a live-cell microscope (Olympus IX81) with controlled temperature (37 °C), CO_2_ (5%) and humidity (100%). Five to ten enteroids/condition were selected at random and every 10 min the enteroids were imaged. The resulting time-lapse was analysed with ImageJ. At every time point, the surface area of the enteroids was measured manually. The relative area increase at every timepoint was then calculated by dividing the area measured at a certain timepoint by the area of the initial state (T = 0) and multiplying by 100. Stimulated enteroids were further incubated for 24 h, upon which the supernatant was collected and the Matrigel dissolved using cell recovery solution as described above. After centrifugation (200 *g*, 5 min, 4 °C) to separate enteroids from the dissolved Matrigel, all fractions were stored at −20 °C.

### IL8 ELISA

To assess the secretion of pro-inflammatory mediators as a response of the enteroids to enterotoxins, an IL8 ELISA (DuoSet, R&D systems) was performed on the collected supernatant and Matrigel domes following the manufacturer’s guidelines. To calculate the IL8 concentration in the samples a calibration curve was fit with Deltasoft software.

### Bacterial adhesion assay

Enteroid monolayers were grown as described above for 5–7 days to 100% confluence. The cells were washed once with sterile PBS at 37 °C and once with culture medium without antibiotics upon inoculation with ETEC. The wild type ETEC strain GIS26 and an F4 deficient mutant strain GIS26*∆faeG* were grown overnight at 37 °C in 5 mL BHI medium while shaking at 180 rpm [29]. Culture density was measured at OD_660_ and verified by overnight plating on BHI agar plates and colony count. After washing with sterile cold PBS, the bacteria were added to the monolayers in culture medium at a multiplicity of infection (MOI) of 10 in duplicate and incubated for 2 h. Next, the excess bacteria were removed by washing the cultures three times with PBS, after which the cells were detached using 0.25% trypsin in PBS for 30 min at 37 °C. Subsequently, a serial dilution was plated on BHI agar and after overnight incubation at 37 °C the number of colony forming units (CFU) were counted.

### Statistical analysis

Statistical analysis was performed with the Kruskal–Wallis Test or the Mann–Whitney *U* test for the independent samples of the ELISA or bacterial adhesion results respectively, in the R 3.4.0 or Prism 6 software package with the significance level set to *p* < 0.05.

## Results

### Porcine small intestinal organoids develop from duodenal, jejunal and ileal crypts

**Figure 1:**
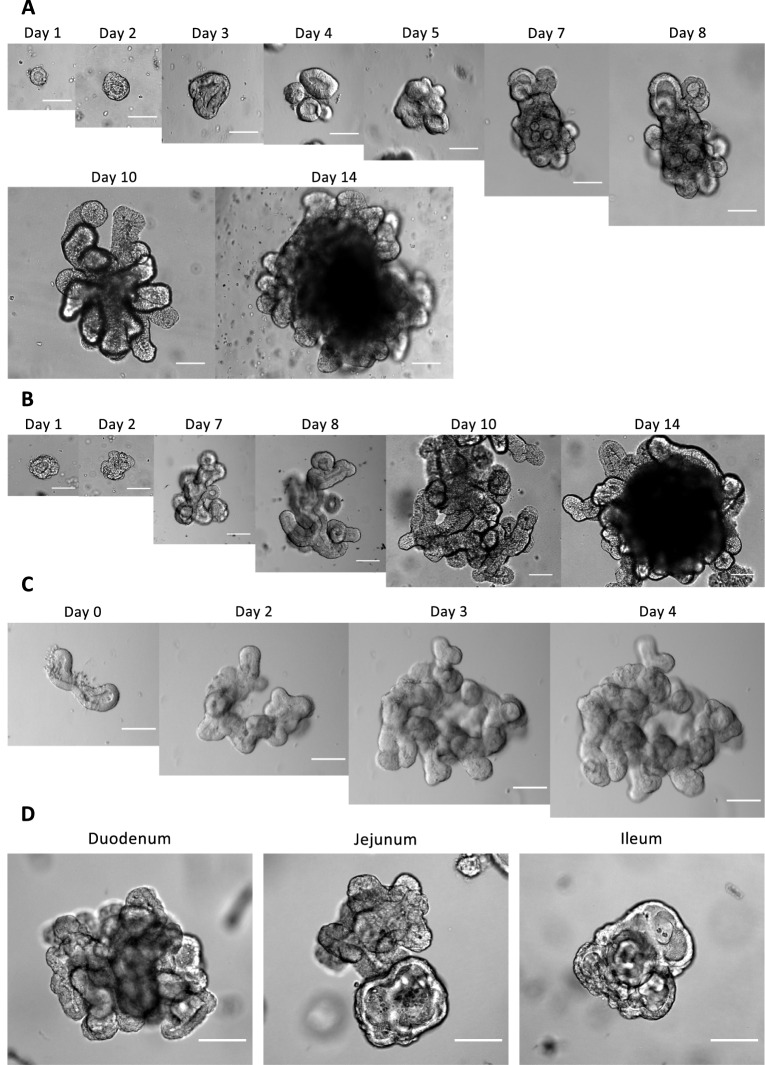
**Development of porcine enteroids from small intestinal crypts.** **A** Enteroids cultured using IESC or (**B**) L-WRN-conditioned IESC medium, respectively. Images were taken from day 1 until day 14 and are representative for enteroid development from jejunal crypts of 9 piglets. **C** Enteroids cultured using Intesticult organoid growth medium were followed from day 0 until day 4 and are representative for enteroid development from jejunal crypts of 4 piglets. **D** Comparison of enteroid cultures originating from duodenal, jejunal and ileal crypts. Images represent enteroids 6 days after passaging and are representative of enteroid cultures from crypts obtained from duodenum (*n* = 4), jejunum (*n* = 10) and ileum (*n* = 8). Scale bar equals 100 µm.

The culture of small intestinal organoids from young and adult pigs was recently developed as a model to study the function of the intestinal epithelium [14, 17]. Now, enteroid cultures are available for many other animal species, including horses, cattle, poultry and dogs [19, 30, 31]. We confirmed the development and growth of porcine enteroids from crypts isolated from the jejunum (Figure 1A). One day after isolation, crypt stem cells developed into small spheroid structures with a pseudo-lumen. These structures grow until day 3–4 when more complex structures begin to form and irregularities or buds are present. These buds further develop into more branched structures until eventually full-grown crypt-like structures can be distinguished. These structures keep growing until day 14 of culture with many crypt-like structures protruding from the central body into the Matrigel matrix. To maintain the enteroid cultures, they were passaged by fragmentation at day 6 or 7 (depending on the development and density of the enteroids). Following this protocol, the enteroids could be cultured for up to 13 passages without any apparent changes in enteroid growth kinetics and morphology.

Recent studies report the use of different culture media to maintain porcine enteroids. Here, we compared IESC medium [14], a commercial Intesticult organoid growth medium [22] and L-WRN-conditioned culture medium to culture and maintain porcine jejunal enteroids [19, 26]. As shown in Figures 1B, C, we confirmed that L-WRN-conditioned IESC medium and Intesticult also supported the development of enteroids from jejunal crypts, closely resembling their development in complete IESC medium. While the enteroids cultured in L-WRN conditioned IESC medium showed similar growth kinetics as those cultured in complete IESC medium, the enteroids cultured in Intesticult displayed an accelerated growth as compared to the other tested media, having crypt-like protrusions after only 4 days post-passage. Of note, jejunal enteroids cultured in Intesticult organoid growth medium did not show any swelling when stimulated with guanylin or enterotoxin-containing bacterial culture supernatant (data not shown). This seems to indicate that Intesticult grown enteroids are less differentiated than the enteroids grown with L-WRN conditioned medium. Therefore, enteroids cultured in L-WRN conditioned medium were used for the functional assays. Previous studies have shown the development of cultures from jejunum and ileum using L-WRN conditioned medium. Here, we show that this medium also supports the growth of duodenal crypts into enteroids (Figure 1D) [17–19, 22].

### Porcine enteroids mimic the response of the small intestine to ETEC-derived enterotoxins

**Figure 2:**
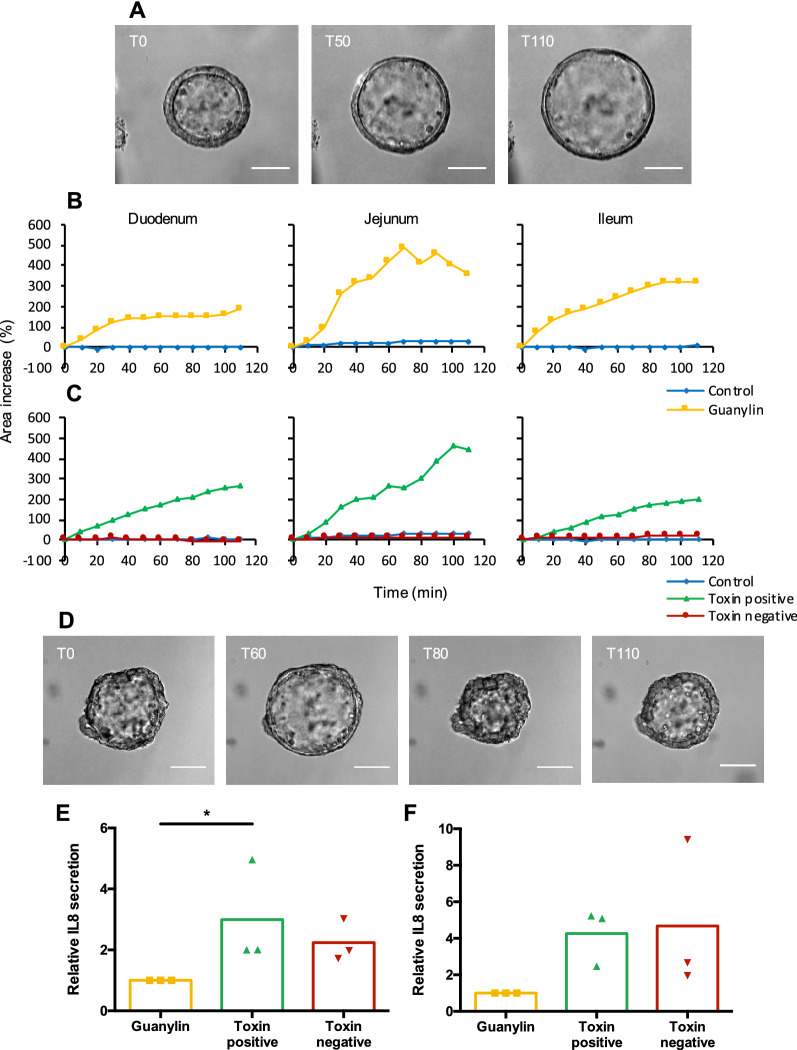
**Porcine enteroids mimic the response of the small intestine to ETEC-derived enterotoxins.** Spheroids derived from duodenum, jejunum and ileum 6 days after passaging were stimulated with enterotoxins or guanylin and imaged using live-cell microscopy. The surface area of the spheroids was measured using ImageJ. **A** Representative images displaying ileal spheroid swelling induced by guanylin (10 µM) at T0, T50 and T110 upon administration. **B**, **C** The average relative area increase of the spheroids was plotted in function of the time after enterotoxin administration. (*n* = 3 for all tissues). **D** Spheroid bursting upon guanylin (10 µM) stimulation. Images are representative for other tissues and swelling inducers. Scale bar = 100 µm. Relative IL8 secretion in medium supernatant (**E**) and Matrigel dome (**F**) of jejunal enteroids stimulated for 24 h with bacterial supernatant with (WT) or without enterotoxins (toxin negative) compared to non-immunogenic guanylin (*n* = 3; Kruskal–Wallis test).

We confirmed that crypts isolated from porcine duodenum, jejunum and ileum develop into complex enteroids similar to previous studies in mice, human and other species [13, 19, 22]. To further evaluate the functionality of the model, the response of these enteroids to enteric pathogens was studied in the context of an infection with ETEC. To this end, the response of porcine enteroids to ETEC-derived enterotoxins, which disrupt the water and electrolyte balance in the gut resulting in fluid secretion to the intestinal lumen, was studied in a swelling assay [6]. The latter is a robust method to assess the ability of the epithelium in the enteroids to secrete and absorb components to and from the pseudo-lumen [32, 33]. To assess if porcine enteroids swell in response to enterotoxin stimulation as well as to evaluate segment-specific responses, an ETEC-derived enterotoxin mixture was added to the enteroid cultures grown from crypts isolated from different parts of the small intestine. After administration, the swelling of individual enteroids was monitored during 120 min using live-cell microscopy. As a positive control, the known secretagogue guanylin of which the STa enterotoxin mimics the function, was added to the cultures. Figure 2A and Additional file 1 clearly show the swelling of an ileal spheroid upon guanylin stimulation. A noteworthy detail is the change in the thickness of the epithelial layer between timepoint 0 and T110. At the latter time point the epithelium is much thinner and probably stretched to its limits. As shown in Figure 2B, spheroids derived from duodenum, jejunum and ileum show a clear and rapid increase in volume in response to stimulation with guanylin as compared to unstimulated enteroids. Similar to guanylin stimulation, spheroids also increased in size upon exposure to ETEC-derived enterotoxins as compared to enteroids stimulated with bacterial culture supernatant obtained from an enterotoxin-negative isogenic ETEC deletion mutant strain. This enterotoxin-mediated swelling was most pronounced in jejunal spheroids (Figure 2C). During the swelling assay however some spheroids suddenly collapse, as the epithelial layer cannot stretch any further and hence ruptures and releases the fluids inside the pseudo-lumen. This so-called bursting can be noticed at 80 min after stimulation of jejunal enteroids (Figure 2D; Additional file 2). Enteroid bursting in swelling assays has also been observed in previous studies with murine and human enteroids [32]. Interestingly, the rupture of the epithelial layer is reversible as enteroids swell again at later time points (Figure 2D).

Previous experiments have shown that porcine intestinal epithelial cells secrete pro-inflammatory mediators upon ETEC infection [9]. To assess if jejunal enteroids respond in a similar manner, they were stimulated with ETEC-derived enterotoxins for 24 h. At this timepoint, the culture medium and the Matrigel dome were collected and the IL8 concentration was determined by ELISA. Bacterial culture supernatant with enterotoxins (WT) triggered IL8 secretion by jejunal enteroids as compared to guanylin stimulation (Figure 2E, F). Bacterial culture supernatant without enterotoxins (toxin negative) did not trigger significant IL8 secretion by the enteroids.

### Development of porcine two-dimensional monolayers from small intestinal organoids

**Figure 3:**
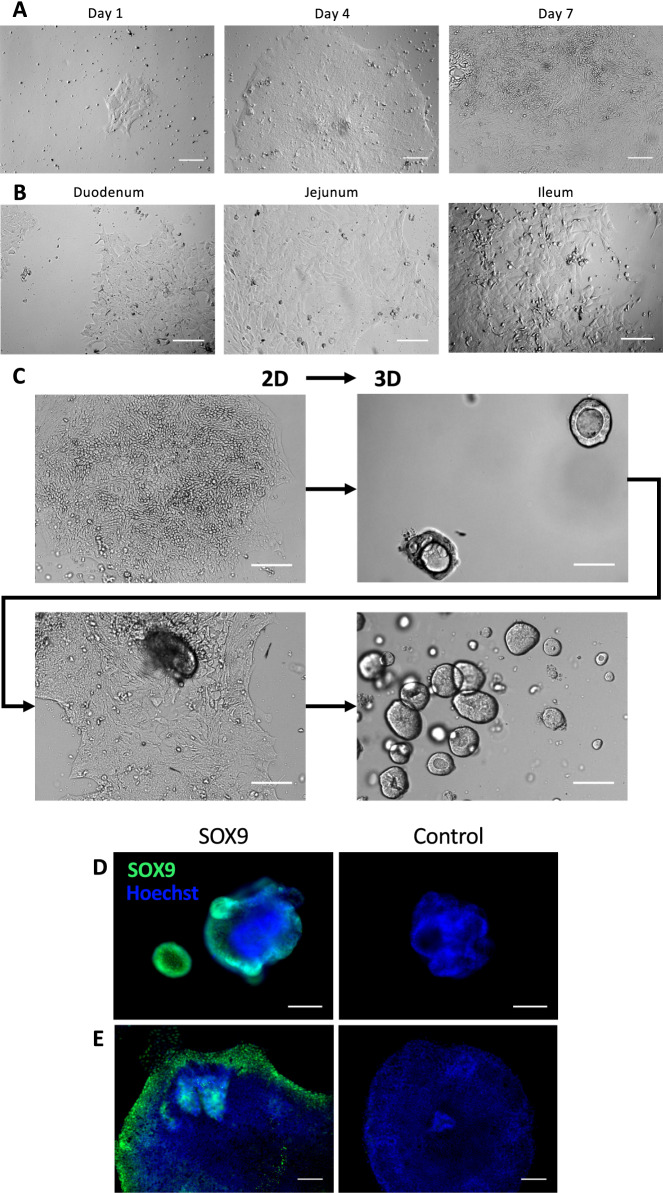
**Porcine enteroid monolayer development and interconvertibility with 3D culture.** **A** Growth of jejunal crypts, plated on collagen-coated wells, using L-WRN-conditioned medium followed from days 1 to 7. **B** Comparison of 2D-monolayers from duodenum, jejunum and ileum at day 3 after passaging. **C** Interchange between 2 and 3D cultures and back of jejunal enteroids representative for 3 piglets. **D** 3D and (**E**) 2D-jejunal enteroid cultures were stained with anti-SOX9 or isotype control antibodies at days 7 and 3 respectively after passage. Images are representative for 2 piglets. Scale bar equals 100 µm.

Despite the many advantages of enteroids, a major limitation of these 3D cultures in the study of host–pathogen interactions is the inaccessibility of the apical surface of the epithelium. To facilitate access to the apical side of the intestinal epithelial cells, 2D-monolayers were developed. Previous studies in murine, human and porcine enteroids have shown that monolayer formation can be achieved on a variety of surface coatings, including Matrigel, collagen and agarose [18, 34]. Here, we assessed the formation of monolayers when seeding single cell suspensions from duodenum, jejunum or ileum on collagen type IV-coated wells. These quickly attach to the collagen-coated surface and form small patches which then further expand outward forming confluent monolayers (Figures 3A and B). Interestingly, cells within these monolayers retained the ability to self-organise into 3D enteroids in Matrigel, even after another round of passaging as monolayers, implying the continued presence of intestinal epithelial stem cells within these cultures (Figure 3C).

To further verify the presence of stem and progenitor cells, we stained jejunal enteroids and monolayers for SOX9, a transcription factor which modulates proliferation and development of these crypt-residing cells (Figure 3D). Although small spheroids almost entirely consist of SOX9^+^ cells, larger spheroids and more complex enteroids show a less pronounced overall *SOX9* expression, but with a distinct localization of SOX9^+^ cells in the buds. When fragmented enteroids were cultured on a collagen-coated surface and developed into monolayers, SOX9^+^ cells were clearly present. These were mainly localized at the edges of the monolayer patches, confirming the outward growth mentioned above (Figure 3E).

**Figure 4:**
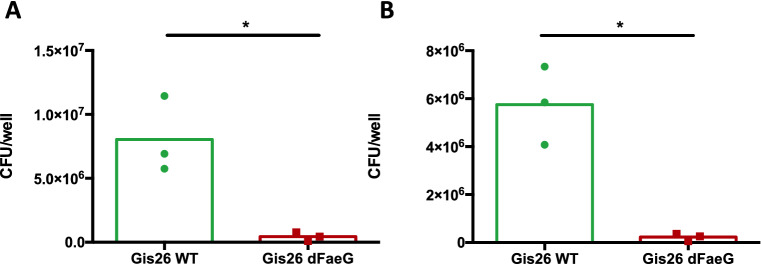
**F4-mediated bacterial adhesion on 2D-enteroid monolayers.** **A** Jejunal and (**B**) ileal monolayers were grown until 100% confluent and infected with ETEC bacteria with (Gis26 WT) or without F4-fimbrae (GIS26*∆faeG*) at a MOI of 10 in duplicate (*n* = 3 for jejunum; *n* = 3 for ileum; Kruskal–Wallis test; * *p* < 0.05).

To address if these monolayers allow adherence of ETEC as shown in traditional cell culture using porcine intestinal epithelial cell lines, like IPEC-J2 [9, 35], confluent enteroid monolayers isolated from jejunum or ileum were inoculated with a wildtype F4-fimbriated ETEC strain or an F4-deficient isogenic mutant strain. As shown in Figure 4, both jejunal (Figure 4A) and ileal enteroid monolayers (Figure 4B) supported F4-mediated adhesion of ETEC bacteria.

## Discussion

Since the discovery of Lgr5 as a marker for intestinal epithelial stem cells and the development of a method to maintain these cells in culture and drive their expansion and differentiation, the use of mini-guts or enteroids in gastrointestinal research is rising [13]. These enteroids are complex 3D structures composed of intestinal epithelial cells and more closely resemble the intestinal architecture and cellular diversity seen in vivo as compared to frequently used intestinal epithelial cell lines, such as the Caco-2 (human) or IPEC-J2 (porcine) cell lines. In contrast to human and mouse, a handful of papers describe the culture and development of porcine enteroids [14, 17–20, 27]. These reports mainly focussed on identifying different cell lineages present in enteroid cultures and the optimization of culture conditions. Some recent studies used enteroids as a new model to study the interaction of pathogens, such as porcine epidemic diarrhoea virus, porcine deltacoronavirus, *Lawsonia intracellularis* and *Toxoplasma gondii,* with the small intestinal epithelium [21–25, 36]. Moreover, in addition to host–pathogen interactions, enteroids provide a valuable model to study stress responses or the effects of dietary components [37–39]. Here, we built upon these findings to enable the use of these enteroids to study the interaction of ETEC with the small intestinal epithelium in piglets. We showed for the first time that porcine enteroids developed from crypts isolated from duodenum, jejunum and ileum in complete IESC, L-WRN-conditioned IESC and Intesticult organoid growth media. Although the commercial Intesticult organoid growth medium supported the culture of porcine duodenal, jejunal and ileal enteroids, in our hands, cultures grown with Intesticult developed twice as fast as compared to the other two culture media. However, enteroids cultured with Intesticult medium did not swell in response to guanylin or enterotoxin stimulation, implying that these Intesticult grown enteroids seem as such less useful to study the interaction of enterotoxins or pathogens with the intestinal epithelium.

Upon infection with ETEC the epithelium releases vast amounts of electrolytes and water elicited by secreted enterotoxins which results in diarrhoea. The responsiveness towards stimulation with ETEC-derived enterotoxins was tested in a swelling assay to further assess the functionality of the model [32, 33, 40]. Enteroids originating from all small intestinal tissues showed an immediate secretory response upon stimulation with guanylin, a known endogenous secretagogue. Moreover, duodenal, jejunal and ileal enteroids displayed a similar swelling in response to stimulation with ETEC culture supernatant containing enterotoxins as compared to supernatant lacking these enterotoxins. Swelling seemed to be most pronounced in jejunal cultures. The apical directed transport of ions seen in these enteroid cultures, confirms the presence of a polarized epithelium, a feature previously reported in human enteroids [41]. Additionally, ETEC-derived enterotoxins elicit their effect via binding to apical receptors. Hence, the observed enteroid swelling is most likely induced by the heat-stable enterotoxins STa and STb. The latter are small peptides (2 and 5 kDa, resp.) as compared to the heat-labile enterotoxin (84 kDa), and are able to pass through the tight junctions and access the apical surface in the pseudolumen of the enteroids [6]. Furthermore, the ETEC strain used in this assay (GIS26) secretes low levels of the heat-labile enterotoxin LT and average to high levels of the heat-stable enterotoxins, implying that the observed swelling is mainly elicited by the heat-stable enterotoxins [42].

The small intestinal epithelium secretes pro-inflammatory mediators, such as IL8, upon ETEC infection [8, 9]. Our data show similar responses in the enteroid model. The cultures showed an upregulated basolateral IL8 secretion when exposed to bacterial supernatant containing enterotoxins. IL8 functions as neutrophil attracting chemokine and neutrophils added to human organoid cultures are attracted to these organoids and even show invasion of the pseudo-lumen [43].

A major disadvantage of enteroids to study host–pathogen interactions is the difficulty to access the apical epithelium. For instance, Derricott et al. used basal-out enteroids to study *Salmonella* Typhimurium infection [36]. Unsurprisingly, *Salmonella* could not invade the enteroids, as this pathogen invades the host at the apical epithelium. A potential solution might be the use of a 2D-monolayer culture. In the current study, monolayers were obtained upon culture of crypts, enteroid fragments, or single cell suspensions from duodenum, jejunum and ileum applied to collagen-coated surfaces. Moreover, 2D-monolayers could be interconverted to 3D cultures and back retaining the ability to self-organise into complex enteroids, similar to murine enteroid cultures [34]. SOX9 primarily marks stem and progenitor cell lineages however around 50% of the Paneth cells also express SOX9 [14, 44]. Fluorescence staining revealed that the monolayers contained SOX9^+^ cells at their edges, suggesting that the monolayer patches grown from enteroid fragments expand from their edge, similar to what has been previously described in primary murine colonic epithelial cell cultures [34]. In contrast, upon passage SOX9^+^ cells appeared scattered throughout the monolayers (data not shown) as recently described for pig ileal enteroids [18]. The use of single cells as compared to crypts or enteroid fragments might explain this different distribution of dividing stem cells. Finally, 2D-enteroid monolayers of jejunal and ileal origin supported the adhesion of ETEC bacteria demonstrated by the strong adhesion of wild type F4^+^ ETEC (GIS26) as compared to the GIS26∆*faeG* strain which does not express F4 fimbriae [29]. These data show that enteroid monolayers are a valuable alternative in bacterial adherence studies.

Now that enteroids have been established for humans, mice and most farm animals, efforts are ongoing to further improve these cultures. One of the main limitations as mentioned earlier is the difficulty to access the apical surface. In addition to monolayers, recently, human and porcine apical-out enteroids were developed where the polarity of the enteroids was reversed and the apical surface is easily accessible [45, 46]. This will facilitate the study of host–pathogen interactions in 3D cultures. The porcine apical-out enteroids were obtained from jejunal tissue and further research should establish these apical-out cultures from the other segments of the small intestine. Another challenge of working with enteroids is the use of Matrigel. The latter is prone to batch-to-batch variability and although it is mainly composed of extracellular matrix proteins, Matrigel also contains a number of growth factors (even in its growth factor reduced format) and other proteins which can affect the development and responses of the enteroids. The use of more defined hydrogels or synthetic scaffolds could solve this issue [47]. Finally, a further optimisation of the enteroid culture would be to include immune cells, stromal cells or microbiota to better mimic the in vivo situation.

Taken together, the induced physiological swelling, the secretion of IL8 and adhesion of bacteria show that enteroids are a valuable and representative model for ETEC infection of the small intestinal epithelium. These infections still represent a considerable disease burden in man and livestock species [7, 48]. Despite extensive research, many molecular mechanisms involved in the interaction between ETEC and the host small intestinal epithelium remain elusive, primarily due to the lack of adequate models for the small intestinal epithelium. The recent development of enteroids offers opportunities to further unravel the molecular crosstalk between ETEC and the small intestinal epithelium. Here, we aimed to demonstrate the utility of porcine small intestinal organoids to study host–pathogen interactions. We showed the development of porcine enteroids from duodenal, jejunal and ileal corroborating previous results [14, 17, 18, 22]. Guanylin and enterotoxin stimulation of enteroid cultures induced physiological fluid secretion seen as swelling and upon stimulation with bacterial supernatant enteroids secrete the chemokine IL8. These findings support data obtained from previous models, such as the IPEC-J2 cell line and perfused intestinal segments [8, 9], and lay the foundation for further research to elucidate the impact of ETEC-derived enterotoxins on the function of the small intestinal epithelium.

## Supplementary Information

**Additional file 1: Porcine ileal spheroid swelling in response to guanylin stimulation.** Spheroids derived from ileum 6 days after passaging were stimulated with guanylin (10 µM) and imaged using live-cell microscopy for 2 h. Scale bar = 100 µm.

**Additional file 2: Porcine duodenal spheroid retains the ability to swell after bursting.** Spheroids derived from duodenum 6 days after passaging were stimulated with guanylin (10 µM) and imaged using live-cell microscopy for 2 h. Scale bar = 100 µm.

## Data Availability

The datasets supporting the conclusions of this article are included within the article and its additional files.
