# Correction to: Porcine small intestinal organoids as a model to explore ETEC–host interactions in the gut

**DOI:** 10.1186/s13567-021-00977-z

**Published:** 2021-08-03

**Authors:** Bjarne Vermeire, Liara M. Gonzalez, Robert J. J. Jansens, Eric Cox, Bert Devriendt

**Affiliations:** 1 Department of Virology, Parasitology, Immunology, Faculty of Veterinary Medicine, Laboratory of Immunology, Ghent University, 9820 Merelbeke, Belgium; 2 Laboratory of Intestinal Regenerative Medicine, College of Veterinary Medicine, NCSU, Raleigh, NC USA

## Correction to: Vet Res (2021) 52:94 https://doi.org/10.1186/s13567-021-00961-7

Following publication of the original article [1], we have been informed that Figure 2B and C needs to be updated. In the midpanel, the y-axis labeling is partially visible but should not have been visible. The updated figure is given below.

**Figure 2:**
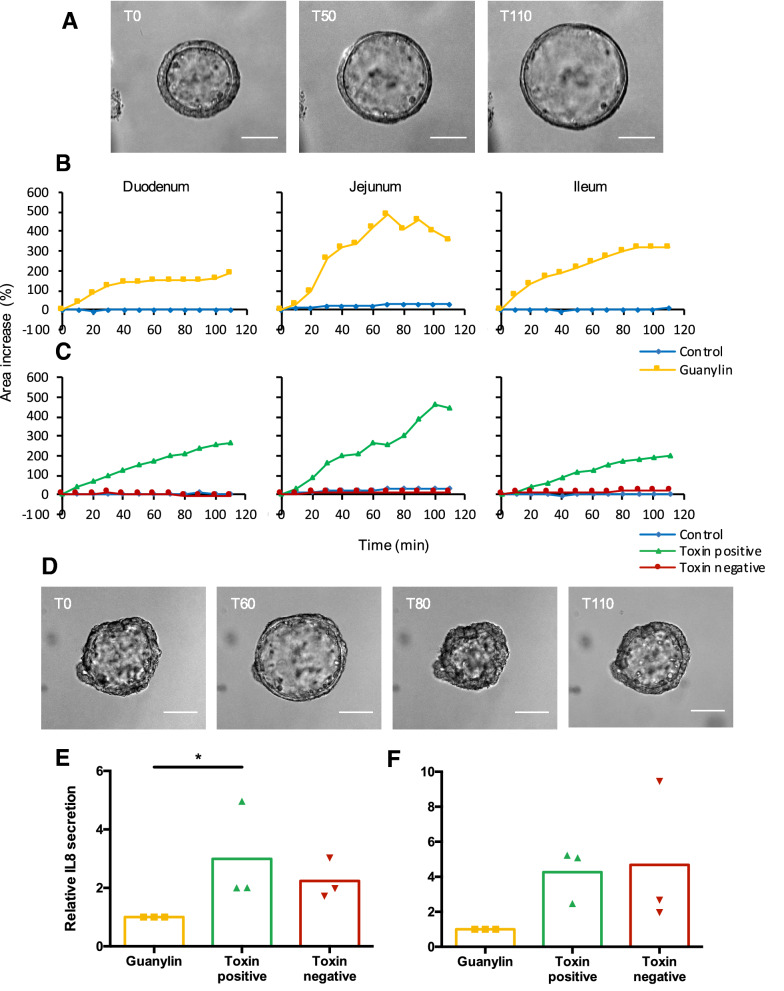
**Porcine enteroids mimic the response of the small intestine to ETEC-derived enterotoxins.** Spheroids derived from duodenum, jejunum and ileum 6 days after passaging were stimulated with enterotoxins or guanylin and imaged using live-cell microscopy. The surface area of the spheroids was measured using ImageJ. **A** Representative images displaying ileal spheroid swelling induced by guanylin (10 µM) at T0, T50 and T110 upon administration. **B**, **C** The average relative area increase of the spheroids was plotted in function of the time after enterotoxin administration. (n = 3 for all tissues). **D** Spheroid bursting upon guanylin (10 µM) stimulation. Images are representative for other tissues and swelling inducers. Scale bar = 100 µm. Relative IL8 secretion in medium supernatant (**E**) and Matrigel dome (**F**) of jejunal enteroids stimulated for 24 h with bacterial supernatant with (WT) or without enterotoxins (toxin negative) compared to non-immunogenic guanylin (n = 3; Kruskal–Wallis test).

The original article has been corrected.
